# Alzheimer’s Disease Seen through the Eye: Ocular Alterations and Neurodegeneration

**DOI:** 10.3390/ijms23052486

**Published:** 2022-02-24

**Authors:** Daniel Romaus-Sanjurjo, Uxía Regueiro, Maite López-López, Laura Vázquez-Vázquez, Alberto Ouro, Isabel Lema, Tomás Sobrino

**Affiliations:** 1 NeuroAging Group (NEURAL), Clinical Neurosciences Research Laboratory (LINC), Health Research Institute of Santiago de Compostela (IDIS), 15706 Santiago de Compostela, Spain; laura.vazquez.vazquez@sergas.es (L.V.-V.); alberto.ouro.villasante@sergas.es (A.O.); tomas.sobrino.moreiras@sergas.es (T.S.); 2 Corneal Neurodegeneration Group (RENOIR), Clinical Neurosciences Research Laboratory (LINC), Health Research Institute of Santiago de Compostela (IDIS), 15706 Santiago de Compostela, Spain; maite.lopez.lopez@sergas.es (M.L.-L.); isabel.lema.gesto@sergas.es (I.L.); 3 Department of Surgery and Medical-Surgical Specialties, Faculty of Optics and Optometry, Universidade de Santiago de Compostela, 15706 Santiago de Compostela, Spain; 4 Instituto Galego de Oftalmoloxía (INGO), Hospital Provincial de Conxo, 15706 Santiago de Compostela, Spain

**Keywords:** Alzheimer’s Disease, amyloid burden, anterior segment, aqueous humor, biomarkers, cornea, posterior segment, retina, tau, tear fluid

## Abstract

Alzheimer’s Disease (AD) is one of the main neurodegenerative diseases worldwide. Unfortunately, AD shares many similarities with other dementias at early stages, which impedes an accurate *premortem* diagnosis. Therefore, it is urgent to find biomarkers to allow for early diagnosis of the disease. There is increasing scientific evidence highlighting the similarities between the eye and other structures of the CNS, suggesting that knowledge acquired in eye research could be useful for research and diagnosis of AD. For example, the retina and optic nerve are considered part of the central nervous system, and their damage can result in retrograde and anterograde axon degeneration, as well as abnormal protein aggregation. In the anterior eye segment, the aqueous humor and tear film may be comparable to the cerebrospinal fluid. Both fluids are enriched with molecules that can be potential neurodegenerative biomarkers. Indeed, the pathophysiology of AD, characterized by cerebral deposits of amyloid-beta (Aβ) and tau protein, is also present in the eyes of AD patients, besides numerous structural and functional changes observed in the structure of the eyes. Therefore, all this evidence suggests that ocular changes have the potential to be used as either predictive values for AD assessment or as diagnostic tools.

## 1. Introduction

Alzheimer’s Disease (AD) is a degenerative disorder of the nervous system with a slow and progressive onset. AD is mainly considered an old-age condition, being the most common neurodegenerative disorder among the elderly in developed countries [1,2,3]. However, based on age at onset, AD can be defined as either early-onset AD (<65 years) or late-onset AD (>65 years) [4]. Overall, early-onset AD is mostly caused by autosomal dominant mutations, with the β-amyloid (Aβ) precursor protein (APP), presenilin 1 (PS1), and presenilin 2 (PS2) genes among the most studied [4]. Remarkably, these mutations collectively represent less than 1% of total cases. In contrast, late-onset AD represents most of AD cases, although its etiology remains unclear because of the multifactorial nature of the disease, where both environmental and genetic risk (e.g., the ε4 allele of APOE (APOEε4)) factors are involved [4,5].

The two pathophysiological hallmarks of AD are neuronal and glial abnormal protein deposits of both extracellular Aβ and intracellular filamentous aggregates of tau [6]. It is well known that such protein accumulations trigger cellular pathways underlying neuronal death but also mediate the activation of microglia and astrocytes, which leads to further damage of surrounding tissues via inflammatory processes [2,6,7]. As a result, there is a progressive atrophy of brain structures, including different lobes (frontal, temporal, and parietal), the entorhinal cortex, amygdala and hippocampus, among others [6,8]. Eventually, these molecular and histopathological changes impact negatively on cortical cognitive functions, such as memory, motor, and language functions, which can promote and/or exacerbate depression or anxiety states [2,3,6]. Although there have been significant advances in the understanding of AD over the last two decades, there are still no reliable treatments to slow down the progression of the disease and/or its onset. Moreover, the lack of an early diagnostic test to accurately determine the onset of AD makes the available treatments almost ineffective, since the neuronal damage is irreparable by the time the disease is diagnosed [9].

Nowadays, the diagnosis of AD is based on clinical, cognitive, and functional criteria using brief cognitive tests (e.g., Mini-Mental Status Examination (MMSE) and clinical dementia rating (CDR)), neuroimaging techniques (e.g., brain scanning by computed tomography (CT), magnetic resonance imaging (MRI), and positron emission tomography (PET)), and biomarker analysis in cerebrospinal fluid (CSF) [10]. Likewise, the National Institute of Aging and the Alzheimer’s Association established complete clinical and cognitive guidelines for the diagnosis of mild cognitive impairment (MCI) or dementia associated with AD, allowing for the classification of individuals with probable AD dementia, possible AD dementia, and probable or possible AD dementia [11]. Early detection/diagnosis is mandatory in order to obtain an effective treatment of AD patients with drugs to delay cognitive loss, as well as with non-pharmacological treatments, such as non-invasive brain stimulation (NIBS), to prolong their quality of life. NIBS approaches are currently getting attention as they show promising results by stimulating different brain regions simultaneously, which improves memory and specific cognitive functions [12,13,14,15,16]. Interestingly, these NIBS approaches could offer a reliable therapeutic option for those AD patients not responding to drug treatments [14].

Therefore, it is important to identify neurodegenerative biomarkers that detect cognitive decline or progression from MCI to dementia [17,18]. In this regard, ophthalmological assessments have detected several ocular changes in patients with central nervous system (CNS) disorders [19]. In many of these disorders, ocular manifestations often precede brain symptoms, suggesting that eye exams could offer an early diagnosis of the underlying disease [19]. Hence, since the eye constitutes an extension of the brain, looking for early ocular manifestations in AD becomes an essential element to explore further.

Several reasons support the viability of the eye as a useful model for the study of AD. First, in comparison with other CNS structures, the eye is relatively accessible for manipulation and in vivo observation. Currently, ophthalmological imaging techniques, such as optical coherence tomography (OCT) or scanning laser ophthalmoscopy (SLO), allow for the visualization and study of the eye by a non-invasive approach [20,21,22]. Secondly, different aspects of visual processing can be affected by AD. Patients with damage in the dorsal region have impaired functions, such as angular discrimination and motion perception, whereas patients with damage in the ventral region have impaired face, color, and shape discrimination [23,24]. Furthermore, it has been suggested that a thinner retinal nerve fiber layer is associated with cognitive decline in subjects with MCI and AD [25]. There are also several studies showing that cognitive decline is associated with brain atrophy and corneal nerve fiber loss [25,26,27,28]. Finally, visual perceptual disturbances are quite common in AD. Loss of visual field, decreased contrast sensitivity, low visual acuity, impaired color vision or motion perception, visuospatial deficits, object agnosia, prosopagnosia, and impaired recognition of emotional facial expressions are some visual deficits that may be involved in AD [29].

Recent evidence suggests a plausible connection between eye alterations and brain changes in AD. Although promising, the usefulness of eye alterations as an early biomarker for AD is still being developed. Here, we review recent advances in the understanding of AD-associated changes in the eye regarding histological structure, Aβ/tau aggregation and vasculature, and their potential to be used as biomarkers in preclinical and clinical AD. Furthermore, we will highlight the caveats of the studies in order to unify consensual criteria that could facilitate the comparison of results between different reports in the future.

## 2. Cognitive Alterations and Visual Repercussions Related to AD

As previously introduced, neuropsychological examination aims to define the state of the different components of a patient’s cognitive status. Cognition is the set of brain-based activities that allow us to be aware of ourselves, others, and our environment [30]. Therefore, the assessment of a potential AD patient should include issues such as memory, orientation, language, behavior, functional ability, executive function, emotional and affective state, and behavioral changes. Several neuropsychological tests, including the MMSE, the CDR, the AD Assessment Scale (ADAS-cog), and the Montreal Cognitive Assessment (MoCA), are used to define the patterns of affected and preserved cognitive abilities [31,32,33]. Importantly, amnesia is the most common form of cognitive impairment attributed to AD, as seen in patients with MCI [34]. However, additional approaches, such as genetics (*APOEε4* carrier), neuroimaging (medial temporal lobe atrophy on MRI, temporoparietal hypometabolism and/or amyloid uptake on PET), and molecular biomarkers (Aβ and tau/p-tau levels in CSF) are also required [35].

Visual perception helps people to acquire information about the surrounding world; when visual perception deteriorates, quality of life worsens and complicates the assessment of other cognitive deficits [29]. AD is not an exception since most AD patients experience defects in visual recognition as a consequence of damage in the associative visual areas [29]. In general, these early visual recognition deficits include impaired ocular fixation and difficulty in visual analysis and synthesis. In particular, such patients often show difficulty in describing the content of a complex photograph, recognizing figures presented from unusual perspectives, or identifying incomplete letters. In advanced stages of AD, patients show visual apperceptive agnosia, involving great difficulty in identifying objects and loss of the ability to reconstruct shapes [29]. In fact, AD patients frequently report difficulty recognizing familiar faces (prosopagnosia) [36]. These patients are also unable to locate objects in space and exhibit a lack of vision–hand coordination (optic ataxia) [37].

## 3. Ocular Alterations Related to AD

**Figure 1:**
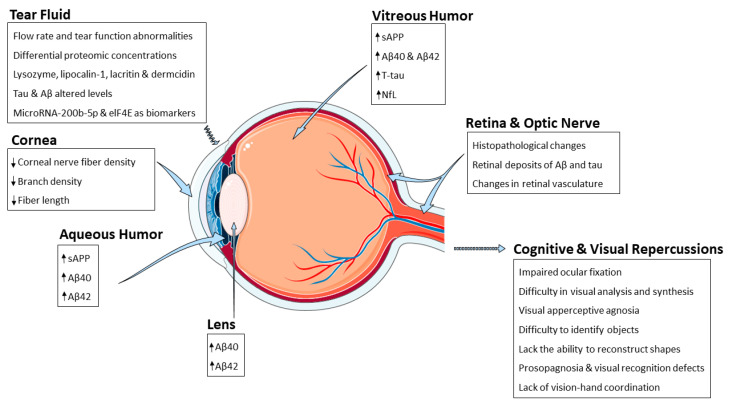
Schematic representation summarizing the AD-related alterations reported so far and the different eye structures affected by such changes. Abbreviations: amyloid-β peptide (Aβ), eukaryotic initiation factor 4 e (eIF4E), neurofilament light chain (NfL), soluble amyloid precursor protein (sAPP).

The eye also displays many features of the AD pathophysiology seen in the brain (Figure 1). In this regard, there are numerous structural and functional changes in the eye linked to AD progression [25,26,27,28]. As mentioned above, irreversible progression, due to the lack of both an effective early diagnosis and a treatment that reverses the AD, exalts the necessity of finding early detection, diagnosis, and curative strategies. In this scenario, eyes could constitute a promising target for new strategies to detect AD progression and onset.

### 3.1. Retina and Optic Nerve

**Figure 2:**
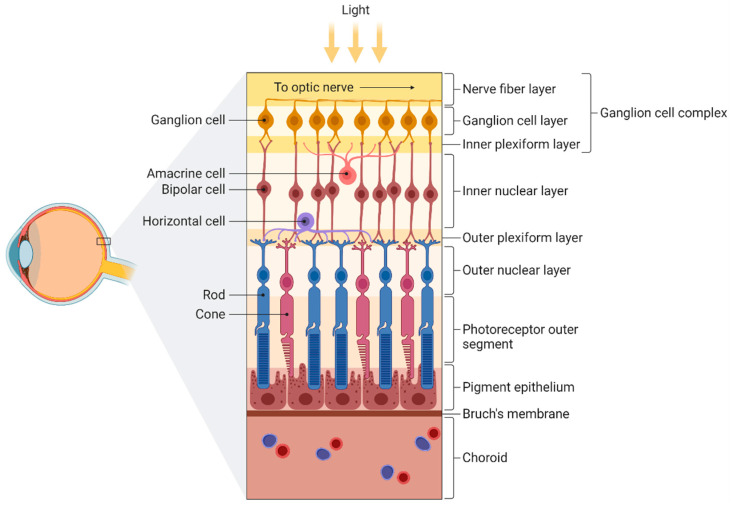
Schematic illustration of the different layers of the retina. From inner to outer: nerve fiber layer (containing axons from ganglion cells), ganglion cell layer (containing ganglion cells), inner plexiform layer (containing dendrites from ganglion, bipolar, and amacrine cells), inner nuclear layer (containing bipolar and amacrine cells), outer plexiform layer (containing extensions from horizontal cells and both rods and cones), outer nuclear layer (containing somas of both rods and cones), photoreceptor outer segment, pigment epithelium, Bruch’s membrane, and choroid. Figure created by BioRender.com software (Toronto, ON, Canada).

The retina is a light-sensitive tissue lining the inner surface of the eye, and it is composed of 10 different layers (from interior to exterior): the inner limiting membrane, retinal nerve fiber layer (RNFL), retinal ganglion cell layer (RGCL), inner plexiform layer (IPL), inner nuclear layer, outer plexiform layer, outer nuclear layer, external limiting membrane, photoreceptor cell layer, and retinal pigment epithelium (Figure 2) [38]. In the central retina, there is a region called the macula that comprises multiple ganglion cell layers and supplies ~50% of the visual input to the cortex [39]. The fovea, a thin retinal zone composed exclusively of cones, is located at the center of the macula and allows for high-acuity vision and color perception [39]. In this review, we will focus mainly on the RNFL, RGCL, and IPL, since these structures form the ganglion cell complex (GCC) and therefore belong to the CNS.

The RNFL consists of axons of the ganglion cells guided to the CNS through the optic nerve (Figure 2). Moreover, there is a type of fiber called the papillomacular bundle, which carries the information that determines visual acuity and radiates from the macula area to the optic disc. The retinal ganglion cells (RGCs) form the RGCL, where they are arranged as a layer with single-cell thickness, except near the macula and the temporal side of the optic disc. Finally, the IPL is a synaptic area where axons from bipolar cells, amacrine cells, and ganglion cells converge. Therefore, the main function of this layer is to integrate inputs from motion detection, brightness changes, and the recognition of contrast and hue.

As a part of the CNS, the retina may also display the classical hallmarks of AD, as previously mentioned. The optic nerve forms a connection between the retina and the brain that may allow the crossing of amyloid precursor protein (APP) from RGCs to the cortex and vice versa. In addition, these changes underlie the atrophy and/or death of different retinal cells, as well as structural and functional modifications in the retinal morphology and vasculature [40,41,42,43]. Considering the retina as a “window” to the brain, its visualization offers a direct and non-invasive approach to detect AD hallmarks without major disturbances in patients.

#### 3.1.1. Histopathological Changes Associated with Progression of Cognitive Impairment 

In the late 1980s, first reports demonstrated degeneration of the optic nerve and RGCs, as well as visual impairments in AD patients [40,41,42,43]. Authors observed a significant degeneration of the optic nerve [40], which was supported by an apparent loss of RGCs [41] in the majority of *postmortem* eyes from AD patients. Besides these histopathological changes, the study of eye movements and visual evoked potentials [42], as well as electroretinograms [43], revealed abnormal patterns in AD patients compared to healthy subjects. A later study also reported impairments in the peripheral vision of AD patients [44]. Altogether, these pioneer studies laid the basics to study retinal degeneration and its relationship with AD. In this regard, a recent morphometric analysis not only confirmed previous studies but also observed different patterns of retinal thinning, with thinner inner layers than outer layers in *postmortem* samples from AD patients [45]. In particular, the RNFL was significantly thinner in the superonasal region than in the superotemporal region, whereas the RGCL displayed a greater reduction in the superotemporal region (remarkable in the macula) than in the superonasal region [45]. Nowadays, novel approaches (e.g., different OCT analyses) have increased knowledge up to the point of exploring the use of the retinal inner-layer thickness as an early biomarker for AD.

Screening work in different populations suggests a potential relationship between the thickness of retinal inner layers and cognitive function [25,46,47,48,49]. The use of magnetic resonance imaging (MRI) in non-dementia subjects revealed a possible link between atrophy in the occipital and temporal lobes and reduced GC-IPL thickness [46], as well as between atrophied visual and limbic networks (hippocampus and cingulum) and thinner RNFL [48]. Interestingly, OCT measurements from preclinical AD patients (neocortical Aβ accumulation, no cognitive impairment) did not reveal significant changes in either pRNFL or retinal inner-layer thickness compared to controls [50,51]. On the contrary, several follow-up studies suggested a relationship between decreased RNFL thickness and cognitive deterioration [25,47]. Indeed, RNFL thinning increases the risk of gradually developing an episodic memory impairment, which is markedly related to the AD progression [47]. Another follow-up study by Mutlu and colleagues [49] reported interesting data that show a probably positive association between GCL-IPL thickness and prevalent dementia; the risk of suffering AD is increased when RNFL thickness is reduced. Prospective studies in preclinical AD patients highlighted the plausible role of RNFL and inner macular layers as early biomarkers. Santos and colleagues [42] reported accelerated macular RNFL (mRNFL) volume loss in preclinical AD patients compared to controls over a 27-month period, which also corresponded to increased cerebral amyloidosis [52]. Moreover, this decrease in mRNFL volume was negatively correlated with total neocortical Aβ accumulation and positively correlated with a loss of sensitivity to audiovisual stimulus [52]. Likewise, another study in preclinical AD patients showed that subjective memory impairment (SMI) and anxiety were positively correlated with mRNFL volume and mRNFL thinning compared to controls 27 months after baseline data [53]. Recently, a study showed that preclinical AD subjects (SMI and brain amyloid aggregation) had significant thickening of their inner nasal macular region at baseline and after 24 months [54].

It is widely accepted that MCI precedes clinical symptoms of AD, representing the first phase of the disease in most cases (see review in [6]). It is well known that MCI patients do not have dementia, but they have an increased risk of developing it in the future [34]. Therefore, studies on subjects with MCI may contribute to finding an early retinal-change warning about the onset of AD. Similar to screenings, OCT recording of patients with MCI displayed an overall diminished peripapillary RNFL (pRNFL) thickness when compared to controls [55,56,57,58,59]; however, the results differ in the specific location where the higher RNFL thinning occurs: all quadrants [55], inferior [56], superior and inferior [57,59], and superior and temporal [58]. In contrast, a few studies did not find significant differences in the pRNFL thickness of MCI subjects compared to controls [60,61,62]. Regarding the thickness of retinal inner layers, several studies obtained contradictory results. The RGCL was thinner in its temporal sector in MCI subjects [63]. The GC-IPL thickness of MCI patients showed a significant reduction in superior, inferior, and nasal sectors [61]; or in the inferior, inferotemporal, and superonasal sectors [60] compared to controls. Similarly, a significant reduction in GCC thickness was observed in MCI patients when compared to control subjects [59,61]. However, another study reported no significant changes in GCC thickness compared to controls [64]. Furthermore, patients with MCI showed other significant changes compared to controls, such as mRNFL thinning in the superonasal sector [64], superior and inferior sectors [61], and temporal and inferotemporal sectors [63]; reduced macular volume [58]; and inner average perifovea thinning [59,61]. Interestingly, Almeida and colleagues [50] revealed significant correlations between MMSE scores and both GC-IPL and GCC thickness data in patients with MCI [61].

It is also interesting to decipher the alterations in the retina of MCI subjects who eventually develop AD in order to establish retinal degeneration as an early biomarker for AD. Some of the previous cross-sectional studies also reported changes between MCI and AD retinas, although with some controversies. Whereas no differences were found in the average pRNFL thickness of MCI subjects compared to patients with prodromic AD, patients with severe AD did show a thinner pRNFL compared to MCI subjects [55,62]. However, another study revealed that subjects with prodromic AD have more pRNFL thinning (superior and inferior quadrants) than MCI subjects (inferior quadrant) when compared to controls [56]. A study in prodromic/moderate AD patients reported that subjects with AD had more reduced pRNFL thickness (superior quadrant) than MCI patients [60]. Moreover, AD patients also had more reduced GC-IPL thickness than MCI subjects in all early treatment of diabetic retinopathy study (ETDRS) sectors when compared to controls [60,65]. Regarding moderate AD cases, AD patients showed more pRNFL thinning in the inferior quadrant than MCI subjects (not significant) compared to controls [58]. In contrast, Tao and colleagues [49] did not find changes in either pRNFL or GCC thickness between AD patients and MCI subjects compared to controls [59]. Unfortunately, there are few longitudinal studies regarding the differences between MCI subjects and AD patients. In a pioneer study, Shi et al. [66] revealed that the inferior quadrant of the pRNFL was significantly thinner in a cohort of patients who changed from healthy to MCI or from MCI to AD compared to subjects whose cognition did not change over 25 months. In a longer follow-up study, Choi and colleagues [56] showed that a decreased GC-IPL thickness (at inferior, inferonasal, and inferotemporal sectors), as well as a lower temporal quadrant pRNFL thickness, was associated with the change from MCI to AD 2 years after baseline data collection [67]. Interestingly, this study also suggested that changes in both GC-IPL thickness and average macula thickness can reveal MCI and AD progression [67].

Since the 2000s, many studies have been performed in order to determine the relationship between retinal degeneration and the progression of AD. In patients with prodromic AD, some studies showed a significantly thinner pRNFL in either the superior quadrant [68] or both the superior and inferior quadrants [69]. On the contrary, other studies revealed no significant differences in pRNFL thickness compared to control subjects [62,70,71,72,73]. Studies in moderate AD subjects also showed some controversial results. An early study reported a significant decrease in pRNFL (in the superior, inferior, and nasal quadrants) from eyes of AD patients compared to controls [74]. Subsequently, other studies found different pRNFL areas significantly affected by moderate AD compared to controls: the temporal quadrant and infero- and superotemporal sectors [75]; the superior quadrant [76]; the superior and inferior quadrants [77,78]; all quadrants [79]; and the inferonasal, inferotemporal, and superotemporal sectors [77]. Although in different quadrants/sectors, all these results point at a clear progressive loss of RGCs underlying AD progression. Furthermore, there is evidence of a negative correlation between pRNFL mean thickness and disease duration since diagnosis [77], as well as a positive correlation between pRNFL mean thickness and cognitive decline [71,78], although with some controversies [72,73]. Interestingly, a prospective study by Trebbastoni and colleagues [60] reported a significant reduction in cognitive decline (from prodromic to moderate AD) and pRNFL thickness (in superior and inferior quadrants) within the AD group [71]. Besides that, increased pRNFL thinning (in the same quadrants) was observed in the AD group compared to controls 12 months after baseline measurements [71].

As previously stated, it has been proposed that the macular region (containing more than 50% of total RGCs) is more sensitive to neurodegenerative processes than the pRNFL region; consequently, it could be a good target to detect early AD-related changes. Interestingly, prodromic AD patients showed significantly decreased mRNFL thickness in fovea, all inner sectors, and the temporal outer sector, as well as reduced macular volume compared to controls [70]. In contrast, recent studies in prodromic AD patients did not find significant differences in the thickness of any retinal inner layer [72,73], although they showed a significant inverse correlation between total macular thickness and brain neurodegeneration (in areas involved in visual processing) by MRI. Additionally, studies in prodromic to moderate AD patients revealed that the whole mRNFL was thinner than that of controls, besides a reduction in GCL thickness [80,81]. A study by Iseri and colleagues [63] was the first to report a correlation between reduced macular volume and lower MMSE score in a cohort of moderate AD cases [74]. Moreover, a reduction in macular thickness was described in six out of nine ETDRS sectors of AD eyes compared to controls; unfortunately, this macular thinning could not be associated with a specific layer due to the low resolution of the OCT device used. Subsequent studies showed higher mRNFL thinning in eight out of nine ETDRS sectors [77,78]. When analyzing the GCC, the results not only showed a significant reduction in the GCC thickness of moderate AD eyes compared to controls [65,75,78,82] but also a negatively significant correlation between GCC thickness measurements and MMSE scores [75,78,82]. Moreover, GCC thinning was positively correlated with a longer AD duration [25]. Importantly, the work of García-Martín and colleagues [75] was first to demonstrate GCL damage in AD retinas, confirming suggestions of previous studies.

Overall, there is no general point of agreement relative to the presence and/or time of retinal histopathological changes. Nevertheless, prospective studies suggests clear association between structural changes and different AD stages. This is remarkable when combining several layers, such as RNFL + GCL, GCL + IPL, or the GCC. To determine whether such changes can be used as an early biomarker of MCI or AD or the conversion from MCI to AD requires further prospective and uniform studies.

#### 3.1.2. Aβ and Tau Accumulation in AD Retinas

As previously mentioned, different visual alterations can be seen at the early stages of AD, many of which underlie neuronal damage in the inner retina, leading to the loss of RGCs and subsequent optic nerve degeneration. Besides the plausible death of RGCs by trans-synaptic retrograde degeneration due to the degeneration of visual-related cerebral structures [40,41,43], an important role for Aβ and tau deposits in the degeneration of inner retinal layers has also been suggested. However, there is controversy as to whether such accumulations are actually relevant and/or related to the retinal AD phenotype.

Studies in *postmortem* AD eyes did not detect abnormal Aβ, either in the retina or the optic nerve [40,41,83,84,85]. Likewise, some studies in animal models of AD agree with these results. Retinas from double transgenic mice (APP/PS1: chimeric mouse/human APP (Mo/HuAPP695swe) and a mutant human presenilin 1 (PS1-dE9)) displayed either minimal or no retinal aberrant Aβ accumulation, even when there was robust expression of APP in all neuronal types [86,87]. Similarly, Schön and colleagues did not observe retinal accumulation of Aβ in P301S transgenic mice (expressing the mutant form of human tau) [85], which was expected to be an animal model for tau pathology, not for Aβ. In contrast, many other postmortem and in vivo studies in animal models disagree with such results. In postmortem AD retinas, the presence of Aβ plaques within inner layers has been observed [76,88,89,90]. Specifically, the RGCL (including melanopsin RGCs) [76,89,90] and both the superior and temporal quadrants [89,90] are the areas with the most significant Aβ accumulation. Recently, another *postmortem* study showed retinal APP/Aβ pathology in AD patients, although it was not enough to discriminate between AD cases and controls [91]. Interestingly, these Aβ accumulations did not display a fibrillary form [91]. Intriguingly, a few studies [88,92,93] did report retinal Aβ plaques in double transgenic mice at similar time points to those used in previously cited works [86,87]. Such studies revealed the existence of Aβ plaques in the RNFL, RGCL, and IPL [88], which even preceded cerebral Aβ deposition [88,92]. Similarly, other results in double transgenic mice also showed aberrant retinal Aβ in same layers previously mentioned but only at later stages [94,95]. Finally, outcomes in the 3xTg-AD mouse model (the only model to exhibit both Aβ and tau pathology) also have some discrepancies. Although the presence of Aβ plaques in the retina was reported in pre-symptomatic 3xTg-AD mice (including RGC death by apoptosis), this Aβ host was not detected in inner retinal layers until approximately 7 weeks of age (middle-aged mice) [96]. However, recent advances have refuted or nuanced these achievements [97,98]. Significantly RGC dendritic loss was detected in 3xTg-AD mice at 12 months of age (old mice) but not at 6–7 months, and it was not accompanied by a reduction in the number of RGCL nuclei [97]. Strikingly, Rodrigues-Neves and colleagues did not find retinal changes regarding Aβ levels, retinal cell death by apoptosis, or decreased RGCs when looking at early stages in 3xTg-AD mice (4–8 months of age) [98].

Besides abnormal Aβ accumulation, the other hallmark of AD is the protein aggregates of hyperphosphorylated tau, which cause most of the pathological features seen in AD [6]. Again, studies in *postmortem* retinal samples from AD patients revealed disparate results, which is in line with the results discussed above. Several studies did not observe either hyperphosphorylated tau or tau aggregates [40,41,83]. Curiously, one study reported that hyperphosphorylated tau (only with the AT8 antibody, which targets the phosphorylation sites Ser202 and Thr205), but no tau aggregates, was detected in the RGCL and IPL of postmortem retinas of AD patients [85]. Similarly, a diffuse pattern of hyperphosphorylated tau expression has been seen with three different antibodies (AT8, AT100 (phosphorylation sites Thr212 and Ser214), and AT270 (phosphorylation site Thr181)) in the retinas of AD patients, with no presence of fibrillar tau [91]. Studies in mouse models of AD reveal differences depending on the strain used. Whereas P301S mice developed early hyperphosphorylation of tau, showing hyperphosphorylated tau in different epitopes, retinal fibrillar aggregates of tau and a tau-mediated axonal transport impairment in RGCs [85,99,100,101]. Results in the double transgenic APP/PS1 mice are controversial. For example, Zhao and colleagues [102] did report an increase in the levels of retinal phosphorylated tau compared to controls, but Chidlow and colleagues [86] could not find the presence of phosphorylated tau in the retina. The fact that these studies used different antibodies to detect phosphorylated tau may be the cause of such disparity. A deeper study performing biochemical techniques in the retinas of triple transgenic mice (3xTg-AD) revealed notable retinal tau accumulation in the soma and dendrites of RGCs, which surprisingly preceded cerebral accumulation [103]. Moreover, the authors did not find tangles of hyperphosphorylated in these mice at 3 or 6 months of age [103]. In contrast, another report showed tangles of hyperphosphorylated tau in the RGCL of young mice by using the same antibody (1.5–2.5 months of age) [96]. More recently, Rodrigues-Neves and colleagues [98] corroborated previous results by reporting an increase in phosphorylated tau at Ser396 in the retina of 3xTg-AD mice at both 4 and 8 months of age. Strikingly, a study revealed that an N-terminal-domain truncated tau is associated with the degeneration of the retinas of Tg2576 transgenic mice (carrying a mutant form of APP linked to early-onset familial AD), highlighting the role of other post-transcriptional modifications of tau during AD progress [104].

In summary, there is no consensus regarding the presence of Aβ and tau deposits in retinas from either animal models or AD patients. Based on the literature, it is plausible that Aβ or tau or both could be found in AD retinas due to seeding [105]. However, the lack of consistent evidence in both animal and clinical research requires further studies to decipher the role of such accumulations in retinal neuronal fate and how this affects the retinal AD phenotype.

#### 3.1.3. Changes in Retinal Vasculature Associated with Progression of Cognitive Impairment 

There is growing evidence that points to a neurovascular component as the trigger for development of AD [7]. Importantly, vascular dysfunction has been suggested to start in the pre-symptomatic stage of the disease [106,107]. Similar to histopathological changes, alterations in the retinal vasculature may underlie AD cerebrovascular pathology; therefore, retinal imaging appears to be a promising approach for characterizing blood vessels in vivo and identifying markers related to the cerebral microvasculature in AD [108].

A massive prospective clinical study (*n* = 5553, 11 years) on the elderly population found that both higher venular calibers and smaller arteriolar calibers are associated with an increased risk of suffering dementia [109]. Studies in preclinical AD subjects also evidenced interesting results. For example, preclinical AD patients (neocortical Aβ accumulation, no cognitive impairment) showed increased retinal arterial pulsation, as well as reduced retinal venular pulsation, with no other changes in systemic vascular parameters correlated with cerebral amyloid burden in those patients [50]. In addition, Aβ+ patients (measured in their CSF) compared to Aβ− subjects (both groups with normal cognition) revealed larger foveal avascular zone (FAZ) [110]. Therefore, FAZ was suggested as a potential tool to detect amyloid plaques in the brain. Recently, another study in preclinical AD patients showed a significantly increased retinal vessel density in the brain compared to healthy controls, which suggests an underlying retinal inflammation during the preclinical AD stage [111]. In contrast, the authors found no significant differences in the FAZ area. Previously, an increased vessel branching asymmetry, as well as a reduction in vessel diameters, was found in neocortical Aβ+ subjects compared to their Aβ− counterparts [112].

In general, retinal vascular measurements do not reveal major changes between MCI and control subjects, which puts into question the results from studies conducted on preclinical AD subjects. No differences were found regarding vessel density, perfusion density, venous diameter, and FAZ area when comparing retinas from MCI subjects with those from healthy subjects [65,113]. Some studies did report a significant decrease in vessel density [114] and retinal blood flow/speed [113,115] in MCI eyes compared to controls.

Differences between MCI and AD patients are consistent with those seen for MCI vs. control subjects. Changes in vessel and perfusion densities are already significantly decreased in prodromic to moderate AD patients compared to MCI subjects, whereas no significant changes were observed in the FAZ area [65]. Furthermore, AD patients showed a decrease in venous diameter and blood flow compared to MCI subjects [113], as well as a reduction in arterial dilatation [81].

Regarding AD retinas, changes were detected very early in the symptomatic onset. Reduced blood flow and impaired tissue perfusion in the inner retinal layers have been shown in prodromic AD cases [115]. Den Haang and colleagues found no significant changes regarding retinal vessel density and FAZ size from prodromic AD retinas compared to control retinas [116]. Nonetheless, some studies reported that prodromic to moderate AD patients display both a reduced vessel density and branching pattern complexity, as well as higher venular tortuosity [114,117,118] or reduced vessel and perfusion densities compared to control measurements [65]; however, the FAZ area remained unchanged [65,117]. Changes in venous diameter, as well as blood flow and speed, were also seen in AD subjects compared to controls [67,113,118]. Interestingly, a study by Jiang and colleagues found a correlation between the loss of retinal vasculature and decreased thickness in the GC-IPL [114]. Similar observations in MCI versus AD subjects, the vasodilatation of arteries from AD eyes was shown to be impaired compared to healthy controls [81]. However, another study found no differences regarding retinal vasodilatation in AD eyes compared to control counterparts [50]. In contrast to previous reports, retinal vascular caliber and branching measurements remained unaltered in moderate AD cases [119]. In later stages of AD, an overall reduced retinal vascular density and an enlarged FAZ area were discovered compared to the control group [120]. Moreover, the authors also found a significant correlation between MMSE scores and both vascular density and FAZ parameters. A recent case report of severe AD revealed a significant loss of retinal vessels, as well as a larger FAZ area [121].

Although reduced cerebral blood flow in brain areas particularly vulnerable to AD is observed in healthy cognitive APOEε4 carriers compared to non-carriers [122], it seems that studies in preclinical or clinical AD patients have not found a similar pattern so far [109,111,116]. However, the number of studies assessing the impact of APOEε4 on retinal vasculature is low; therefore, more investigations are needed to decipher whether retinal scans can be useful for finding reduced/changed retinal vascular parameters related to AD in APOEε4 carriers.

To summarize, there is controversy about the relationship between several vascular changes and their correlation with AD development. Moreover, results do not yet point to feasible vascular measurements to distinguish between MCI and healthy subjects. However, some vascular changes from MCI to AD retinas are becoming clear. Therefore, more research in preclinical AD patients is mandatory to refine the findings of new and more specific vascular alterations linked to AD progression.

### 3.2. Tear Fluid

The analysis of different body fluids is a strategy to elucidate the pathophysiological mechanisms underlying a wide variety of diseases. Remarkably, the rising incidence of AD has highlighted the necessity to develop new screening and early-diagnosis techniques using less invasive and cheaper methods. In this regard, tear fluid analysis has become a promising non-invasive alternative in the search for biomarkers associated with AD.

**Figure 3:**
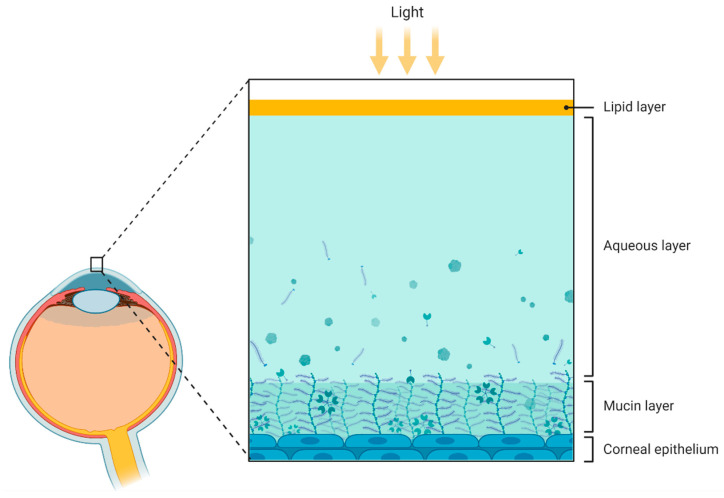
Schematic representation of the compartments presented in the tear fluid. From outer to inner: lipid layer, aqueous layer, mucin layer (containing glycoproteins called mucins), and corneal epithelium. Figure created by BioRender.com software (Toronto, ON, Canada).

Tear fluid consists of an aqueous–lipidic layer containing proteins, mucins, lipids, water, and electrolytes (Figure 3). It covers and nourishes the ocular surface, playing a key role in protection against external pathogens. Even though the volume of tear samples is small, advances in proteomics and lipidomics have allowed for a better understanding of tear components and their involvement in different diseases [123]. In this regard, biotechnological tools are being developed to optimize the extraction of samples from collection devices (such as Schirmer strips or capillaries) and to improve the detection of markers, such as β-amyloid, in tears (using biosensors) [124]. Hopefully, these new tools will help to overcome the limitations related to the small volume of tear samples. To date, there are already numerous ocular (e.g., keratoconus, dry eye, and glaucoma) and systemic conditions (such as diabetes mellitus, thyroid dysfunction, and neurological diseases) that can be detected and evaluated by biomarkers at the lacrimal level [125,126].

Modifications in total tear proteomic concentration, as well as abnormalities in flow rate and tear function, have been reported in AD, suggesting a dysfunction in the autonomic nervous system [127] (Table 1). Indeed, significantly decreased levels of lysozyme, lipocalin-1, and lacritin, as well as increased levels of dermcidin, were found to be expressed in the tear fluid of AD patients [128] (Table 1). Remarkably, the combination of all of these factors was demonstrated as a potential AD biomarker, as they showed a sensitivity of 81% and a specificity of 77% for prediction of the disease [128]. Likewise, CSF biomarkers were measured in tear samples to assess a new non-invasive tool to predict and detect AD. Specifically, three Aβ peptides (Aβ38, Aβ40, and Aβ42), total tau (t-tau), and phosphorylated tau (p-tau) were analyzed in patients diagnosed with clinical dementia, MCI, and subjective cognitive decline (SCD), as well as in a control group [129] (Table 1). Interestingly, the concentrations of all peptides were higher in tear samples of all patient groups compared to healthy subjects; however, non-statistical differences were obtained. In addition, t-tau levels were significantly higher in dementia, MCI, and SCD compared to the control group [129]. Importantly, p-tau was also detected in all patient groups but not in control subjects. Both t-tau and p-tau showed no differential expression among the three patient groups [129]. Moreover, the association of tear amyloid and tau levels with AD severity and neurodegeneration was also studied, highlighting the potential of tau and Aβ proteins in tear fluid as markers of AD severity [130,131]. In this regard, a recent study also suggested the potential of t-tau and Aβ42 markers at the lacrimal level for the diagnosis/discrimination of AD [130,131] (Table 1). The authors determined a gradual increase in both biomarkers in tears throughout cognitive decline [130,131]. Additionally, Kenny and colleagues observed that the total tear concentration of microRNA-200b-5p was highly upregulated in AD samples compared to the control group [132]. Interestingly, the elongation initiation factor 4E (eIF4E), a polypeptide involved in some cellular processes (e.g., protein synthesis, mRNA stability, and RNA nuclear export), was exclusively detected in the tear samples from AD patients [132] (Table 1). Further studies are needed to elucidate the biological relationship between AD and biomarkers microRNA-200b-5p and eIF4E. In summary, different studies have been pointed at the relationship between tears and CSF to detect potential biomarkers; nonetheless, more studies should address this relationship.

### 3.3. Cornea

**Figure 4:**
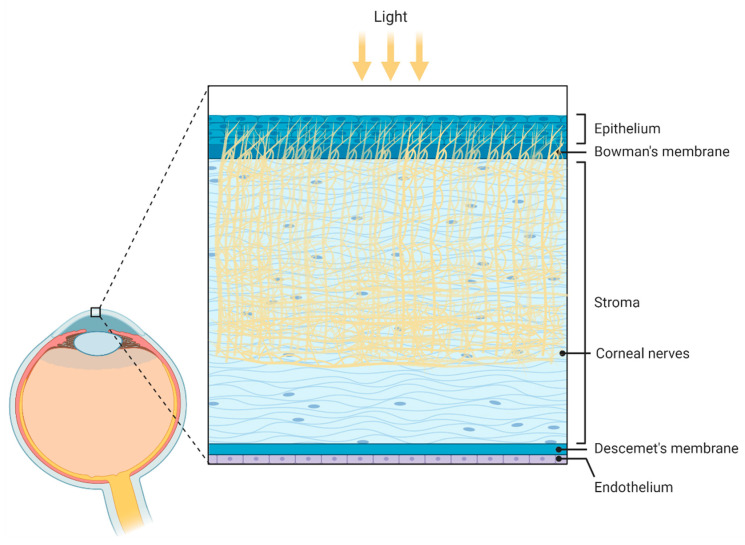
Schematic illustration of the corneal anatomy showing its different layers and structures. From outer to inner: epithelium, Bowmans’s membrane (containing collagen fibers), stroma (containing corneal innervation), Descemet’s membrane, and endothelium. Figure created by BioRender.com software (Toronto, ON, Canada).

The cornea is an avascular and complex tissue comprised of several layers, such as the epithelium, epithelial basement membrane, Bowman’s layer, stroma, Descemet membrane, and endothelium (Figure 4) [133,134]. It is well known that the cornea must maintain transparency to benefit the eye’s refractive capacity.

The cornea is considered the most sensitive structure in the human body because it contains a higher number of nerve fibers [135]. Curiously, a density of 605.8 terminals/mm^2^ was observed in the suprabasal layer of the central corneal epithelium [136], with a corneal innervation 400 times greater than skin innervation and 40 times greater than dental pulp innervation [137]. Activation of the corneal nerves leads to reflex activation of blinking and tearing and contributes to both the inflammatory response and the release of trophic factors.

The peripheral sensory terminals of the corneal nerves are densely integrated in the epithelium and are responsible for the maintenance and preservation of the corneal tissue and tear film [135]. Changes in the morphology and function of the corneal nerves in response to disease, surgery, and aging have been well documented [16,138,139]. In this regard, a wide variety of diseases, including AD, trigger negative ocular surface changes that may be linked to the harmful effects of corneal nerve dysfunction [16,140]. Specifically, loss of corneal nerve fibers was observed in patients with Parkinson’s disease, amyotrophic lateral sclerosis, multiple sclerosis, and MCI or dementia [26,138,139]. In AD, the morphology of corneal nerves has been studied, evidencing corneal nerve degeneration.

Ponirakis and colleagues observed a progressive and significant reduction in corneal nerve fiber density, branch density, and fiber length by corneal confocal microscopy in both MCI and dementia patients compared to age-matched healthy controls [26]. Furthermore, this study also showed that all these corneal nerve fiber measurements were positively associated with cognitive function and functional independence in MCI and dementia patients [26].

One of the weaknesses of studying corneal innervation in patients with AD is the limited range of the techniques used to assess the progression of corneal neuropathy. Corneal nerve morphology is evaluated by non-invasive ophthalmic confocal microscopy, and function and sensitivity are mainly tested using aesthesiometers [141,142]. Moreover, larger studies are needed to establish the diagnostic and prognostic utility of corneal confocal microscopy in people with MCI and dementia. Currently, the use of these tools is mostly reserved for research, and their use in clinical practice is not widespread. Therefore, it is urgent to develop better tools, as well as to refine current ones, in order to use loss of corneal innervation as an early biomarker of AD [143].

On the other hand, molecular changes in corneal tissue could occur together with the loss of corneal nerve fibers and neurological degeneration in AD. Aging-associated imbalance in the homeostatic levels of neuronal-synapse neurotransmitters (such as glutamate, GABA, and acetylcholine) is associated with the cognitive impairment of several neurological pathologies [144]. In this line, reduced levels of acetylcholine at the brain level have been found in AD, contributing to weakening synaptic plasticity [145]. Reduced acetylcholine levels could be compensated by central anticholinesterase drugs that prevent acetylcholine hydrolysis and increase its availability at the synapse. Regarding the cornea, it contains one of the highest concentrations of acetylcholine [146]. In fact, this neurotransmitter is essential for corneal epithelium development and maintenance. As a result, measuring acetylcholine in the cornea of AD patients could be critical; however, no data exists to date. Currently, anticholinesterase drugs are considered the mainstay treatment for AD, although their use is limited to treating symptomatic alterations associated with memory deficiency [145].

Amyloid deposition in corneal tissue has been observed in some corneal diseases, including hereditary reticular corneal dystrophy [147]. Regarding AD, there is still a scarcity of information about the presence and concentration of amyloid and tau in the cornea. Until now, studies carried out on this topic seem to have been focused on animal models. In this line, transgenic mice showed a high cytoplasmic expression of APP and possibly Aβ in the corneal epithelium compared to wild-type controls (using mice that express the human mutation APPswe) [87]. Likewise, APP transcripts in human corneas and in corneas of transgenic mice with AD appear to be longer and more damaging than those expressed in the brain and retina [148].

Given the importance of Aβ deposition in AD, accounting for the role of APP, and knowing that the cornea represents an interesting structure to analyze due to its easy access, studies in humans are mandatory to evaluate predictive AD biomarkers that may be present in AD corneas—not only Aβ, but also others, such as tau protein, nerve growth factor (NGF), and acetylcholine.

### 3.4. Lens

The crystalline lens has been the focus of most research on the anterior eye and AD [146], considered an ideal ocular structure for the detection of Aβ deposits. Expression of APP and Aβ in cultured crystalline lenses has been found in animal studies, suggesting that pathological mechanisms associated with AD may be linked to the development of age-related cataracts [149]. The lens must be optically transparent to refract light on the retina, so the accumulation of protein aggregates over time leads to vision loss and worsening visual perception.

A study of Aβ40 and Aβ42 accumulation in human lenses found that both proteins show concentrations comparable to those in the brain of people with and without AD [150]. Furthermore, both Aβ40 and Aβ42 were accumulated in the cytoplasm of supranuclear/deep cortical lens fiber cells of AD patients, suggesting that Aβ may promote regionally specific lens protein aggregation and supranuclear cataracts [150]. Even though supranuclear cataracts are more common in patients with AD, measuring cataract severity by lens opacity seems a useless non-invasive test for determining the likelihood of developing AD [151]. Kerbage and colleagues carried out a promising in vivo study in a small cohort of AD patients, which aimed to measure Aβ aggregates in the supranuclear region of the lens by using a laser scanning device called a fluorescent-ligand eye-scanning system (FLESS) [152]. The FLESS was designed to detect and measure the emitted fluorescence signal of the fluorescent ligand bound to Aβ aggregates in the supranuclear region of the lens, showing that lens measurements are correlated significantly with F^18^-PET amyloid brain analysis [152]. Although the presence of Aβ in the lens is widely supported by proteomics [150,153,154], more research in preclinical AD patients is needed to confirm whether Aβ accumulation could be used as a prognostic tool [155,156].

### 3.5. Aqueous and Vitreous Humor

Aqueous humor (AH) is an important ultrafiltered blood biological fluid produced in the epithelium of the ciliary body. It is composed of proteins, electrolytes, solutes, and growth factors. AH provides nutrition to the cornea and crystalline lens and removes their excretory metabolic products [157]. It is essential for maintenance of normal intraocular pressure and provides suitable eye shape and optical properties for the eye. Regarding vitreous humor (VH), it is a gelatinous mass that fills the space between the retina and the lens. It directly contacts and acts as an interface between the retina, the lens, and the ciliary body, contributing to the diffusion of a wide variety of compounds [158]. Moreover, proteomic analysis showed that the surrounding tissues influence the composition of the different vitreous regions [158]. Importantly, there is compelling evidence of AD-related ocular changes in both AH and VH levels of several biomarkers [150,156,159,160]. For example, Prakasam and colleagues observed that soluble APP (sAPP) concentration, which is mainly secreted by the retina, was particularly high in VH in comparison with AH; likewise, Aβ40 and Aβ42 concentrations were 50% lower in AH than in VH [160]. Goldstein and colleagues also described that Aβ40 and Aβ42 levels were detectable and measurable in AH, being comparable to those in CSF [150]. In addition, Aβ40 and Aβ42 concentrations in VH were associated with lower cognitive function based on MMSE scores [161]. Regarding t-tau, it was detected in VH and correlated with lower cognitive function, in agreement with findings from studies carried out in CSF [161]. Finally, Aβ40, Aβ42, and t-tau in VH were positively correlated with levels of neurofilament light chain (NfL), a neurofilament subunit also identified in vitreous samples [162]. NfL proteins are found in the cytoplasm of neurons and help to maintain structural stability, neuronal integrity, and impulse velocity. NfL is persistently released at low levels in normal circumstances, but higher levels of NfL were seen in both CSF and blood of AD patients [163]. Although NfL is not currently used as a screening tool in clinical practice, it may be in the future.

## 4. Limitations

It is necessary to consider the significant limitations and controversial results that we find in the majority of the current bibliography, preventing a feasible comparison between studies. For example, some ocular events related to AD are also shared by other ocular neurodegenerative pathologies, such as age-related macular degeneration (AMD) or glaucoma [164]. In fact, some risk factors and pathophysiological mechanisms, as well as aging and histopathological changes, are common features in all of these conditions. For this reason, it would be mandatory to select precisely all the subjects undergoing these types of studies in order to minimize the impact of those diseases on the pathological ocular manifestations seen in the results. However, many studies do not consider some of these aspects, including significant differences in the age of participants between each condition; therefore, results are biased and not reliable for future clinical applications. Another important unsolved question is to fully understand and define the etiology of AD. Although MCI is considered the previous stage before AD development, it is a complex and heterogeneous group that comprises subjects who may not progress to AD but develop non-AD dementia or maintain MCI as part of the normal aging process. Moreover, many studies define the MCI group as cognitively normal (e.g., MMSE scores similar to control scores), whereas others include MCI subjects already showing impaired cognition (significantly lower MMSE score than controls). This corrupts the results, as these MCI cases with altered cognition already display early neuropathological changes of AD. Similarly, a standardized protocol must be established regarding the neuropsychological tools used to assess the grade of dementia in such studies. Each one study different scores and tests to define the level of dementia within groups (e.g., AD and MCI patients), which makes it difficult to compare results between studies. The well-defined guidelines from the National Institute of Aging and the Alzheimer’s Association could be established as the standard protocol to classify individuals across different studies.

Although the examination of different structures of the eye is an interesting field for the development of a non-invasive diagnostic tool, the available imaging tools should be improved for both the analysis of the anterior segment and the study of histologic changes in order to increase the accuracy of the results. Moreover, many measurements of the ocular vasculature may be affected by unrelated AD variables, such as heart rate, arterial pressure, and cardiac cycle. Unfortunately, data measurements from different imaging devices (e.g., two different types of OCT devices) cannot be fully compared, as their settings are not the same. All of this provokes the appearance of reproducibility issues, which limits the comparison of results from different labs and conclusions concerning clinical applications of the concepts.

Finally, protocols for the analysis of Aβ and tau retinal accumulation need major revision and standardization. The controversial results seen in human samples are partially due to a lack of a standard protocol for staining and tissue-mounting procedures. Thus, differences in antibodies and their dilutions, studied histological regions and their sectioning planes, and the postmortem interval influence the variability of results. Importantly, the current animal models available for AD do not recapitulate the full pathology seen in human patients. Furthermore, there is a variability in the strains of AD transgenic mice, even between populations from the same original strain, as well as different phenotypes depending on the sex of the animal (e.g., male 3xTg-AD mice develop a slower and milder phenotype than female counterparts). That is why evidence from animal models should be analyzed cautiously before drawing conclusions abouts its potential use in clinics.

## 5. Concluding Remarks and Future Directions

The main goal of this review was to highlight the role of ocular alterations and protein levels of ocular fluids as potential biomarkers or therapeutic targets in AD. We compiled the most recent studies about eye and ocular alterations during AD progression. Optical examination in patients with neurodegenerative disorders is an emerging field that needs more investigation. Most of the current bibliography in this field is focused on the study of the posterior segment, which includes the retina and optic nerve. In contrast, the anterior structures have received less attention. This is surprising, given several findings suggest that the tear film, cornea, aqueous humor, and lens are also affected in AD and play a prominent role in disease progression. However, many mechanisms and events are not fully understood; therefore, more studies are needed to elucidate the underlying pathways.

Overall, despite controversy, ocular alterations associated with AD are undeniable at both structural and fluid levels. Currently, the lack of protocol standardization and uniform studies are preventing the clinical application of several interesting findings. However, AD-associated ocular changes have an enormous potential to become a non-invasive tool for early diagnosis of AD.

## Figures and Tables

**Table 1:** Studies observing changes in tear proteomic/molecular concentrations.

Publication	Study	Results
Kalló et al. [128]	Examination of changes in tear protein composition from patients with AD compared to control	− Decreased levels of lysozyme, lipocalin-1, and lacritin in AD patients. − Increased levels of dermcidin. − Detecting all these alterations together in preclinical AD subjects might be used as a biomarker to further explore other typical AD measurements (imaging, neuropsychological testing, and CSF analyses.
Gijs et al. [129]	Examination of AD-specific biomarkers in tear fluid from SCD, MCI, and AD patients	− Increased levels of Aβ38, Aβ40, Aβ42, t-tau and p-tau in all conditions compared to controls. − Only t-tau level changes were significantly increased when comparing each condition to controls. − The presence of Aβ peptides, t-tau, and p-tau was shown in tear fluid for the first time.
Gijs et al. [130]	Testing the diagnostic potential of tears as a source of AD biomarkers	− Levels of both t-tau and Aβ42 are positively correlated with the AD stage. − Between disease conditions, t-tau in tears of AD patients was significantly higher than in those of SCI and MCI patients.
Gijs et al. [131]	Observational study to investigate AD-specific biomarkers in tear fluid	− Elevated levels of Aβ40 and t-tau in tear fluid from patients with cognitive impairment associated with disease severity. − Correlated levels of AD biomarkers in tear fluid and CSF from AD patients.
Kenny et al. [132]	Examination of tear fluid to discover disease-specific protein and microRNA-based biomarkers for AD	− eIF4E was present only in AD samples. − Higher abundance of total microRNA in tears from AD patients compared to controls. − Interestingly, microRNA-200b-5p could be used as a biomarker for AD.

## Data Availability

Not applicable.
